# Identification of key genes controlling soluble sugar and glucosinolate biosynthesis in Chinese cabbage by integrating metabolome and genome-wide transcriptome analysis

**DOI:** 10.3389/fpls.2022.1043489

**Published:** 2022-11-25

**Authors:** Lixia Wang, Shu Zhang, Jingjuan Li, Yihui Zhang, Dandan Zhou, Cheng Li, Lilong He, Huayin Li, Fengde Wang, Jianwei Gao

**Affiliations:** ^1^ Institute of Vegetables, Shandong Academy of Agricultural Sciences, Jinan, China; ^2^ College of Life Sciences, Shandong Normal University, Jinan, China

**Keywords:** soluble sugar, glucosinolate, metabolome analysis, transcriptome analysis, Chinese cabbage

## Abstract

**Introduction:**

Soluble sugar and glucosinolate are essential components that determine the flavor of Chinese cabbage and consumer preferences. However, the underlying regulatory networks that modulate the biosynthesis of soluble sugar and glucosinolate in Chinese cabbage remain largely unknown.

**Methods:**

The glucosinolate and carotene content in yellow inner-leaf Chinese cabbage were observed, followed by the combination of metabolome and transcriptome analysis to explore the metabolic basis of glucosinolate and soluble sugar.

**Results:**

This study observed high glucosinolate and carotene content in yellow inner-leaf Chinese cabbage, which showed a lower soluble sugar content. The differences between the yellow and the white inner-leaf Chinese cabbage were compared using the untargeted metabonomic and transcriptomic analyses in six cultivars of Chinese cabbage to explore the metabolic basis of glucosinolate and soluble sugar. Aliphatic glucosinolate and two soluble sugars (fructose and glucose) were the key metabolites that caused the difference in Chinese cabbage’s glucosinolate and soluble sugar. By integrating soluble sugar and glucosinolate-associated metabolism and transcriptome data, we indicated *BraA05gAOP1* and *BraA04gAOP4*, *BraA03gHT7* and *BraA01gHT4* were the glucosinolates and soluble sugar biosynthesis structural genes. Moreover, *BraA01gCHR11* and *BraA07gSCL1* were two vital transcription factors that regulate soluble sugar and glucosinolate biosynthesis.

**Discussion:**

These findings provide novel insights into glucosinolate and soluble sugar biosynthesis and a possible explanation for the significant difference in nutrients between yellow and white inner-leaf Chinese cabbage. Moreover, it will facilitate genetic modification to improve the Chinese cabbage’s nutritional and health values.

## Introduction

Chinese cabbage is one of the essential vegetables in the world. They are the most widely grown vegetables in China and northern areas and account for over one-quarter of the total annual vegetable consumption. The leafy head comprising numerous incurved leaves is the main edible organ. Soluble sugar, carotenoid and glucosinolate are the most common nutrient compounds affecting the flavor of Chinese cabbage (Ishida et al., 2014; Cao et al., 2021; Cheng et al., 2022). However, over the past few decades, intensive breeding has been mainly focused on yield and disease resistance rather than the quality of vegetables. With the increase in citizens’ living standards, the market-driven orientation artificial selection strategy becomes more critical. Furthermore, functional Chinese cabbage has gained popularity within health and wellness circles in recent years with the increasing diversity. Therefore, Chinese cabbage has been the subject of much research to evaluate its nutrient compounds and characteristic flavors (Higdon et al., 2007).

Glucosinolate is sulfur- and nitrogen-containing plant secondary metabolite found in cruciferous vegetables, whose hydrolysis products contribute to the unique flavours and tastes of Brassica species (Shim et al., 2016; Bischoff, 2021). Glucosinolate is known for its beneficial effect on human health, such as strong anti-cancer effects and regulatory functions in inflammation and dampening the stress responses and antimicrobial properties (Hu et al., 2022; Jo et al., 2022; Tandayu et al., 2022). Aliphatic glucosinolate has been reported as the primary type of glucosinolate in *Brassica rapa* (Liao, 2011). Interestingly, aliphatic glucosinolate, positively correlated with carotenoid content in Chinese cabbage, indicated the synergistic regulation between these nutrient components (Baek et al., 2016). Content and biosynthesis of glucosinolate that affects fruit flavor have been widely studied in model *Arabidopsis* (Harun et al., 2020), which paved the way for elucidating the molecular mechanism of glucosinolate biosynthesis in Chinese cabbage. The biosynthesis of aliphatic glucosinolate originates from the elongation of methionine (Met). *AOPs (*2-oxoglutarate-dependent dioxygenases*)*, *SUR1(*C-S lyase*)*, *UGT74 (*UDP-glycosyltransferases*), BCATs (*branched-chain amino acid aminotransferase*)*, *CYP79s* (cytochrome P450 monooxygenases) and *CYP83s* (Kang et al., 2018; Zuluaga et al., 2019), *MAM* (Methylthioalkylmalate synthase) (Das, 2021), *SOT* (sulfotransferases) (Klein and Papenbrock, 2008) play a vital role in glucosinolate biosynthesis. Overexpression of *CYP79s* and *CYP83s*, which are involved in forming the core glucosinolate structure, increased the aliphatic glucosinolate level in Chinese cabbage (Zang et al., 2008). In addition, transcription factors, such as MYB, ERF and bHLH, are all involved in the regulation of glucosinolate biosynthesis (Chhajed et al., 2020; Mitreiter and Gigolashvili, 2021). In the previous report, *MYB122* (transcription factor), *CYP79B2*, *UGT74B1*, *SUR1*, *SOT16*, *SOT17*, *SOT18* (core structure biosynthesis genes), and IGMT1 (indole glucosinolate side chain modification gene) play a differentiated role in the transcriptional response pattern of JA (jasmonic acid) in two broccoli cultivars (Ku et al., 2016). Therefore, these metabolite and transcript biomarkers could be helpful in an effective marker-assisted breeding strategy for improving the resistance in Brassica vegetables. Another study indicated three *MYB28* homologs regulating glucosinolate biosynthesis in Chinese kale sprouts (Guo et al., 2016).

Soluble sugar, including sucrose, glucose and fructose, is one of the essential qualitative traits to evaluate the shelf-life of Chinese cabbage and is also the vital element that provides energy and bare carbon skeletons for various metabolic pathways (Shen et al., 2018; Liu et al., 2020). In addition, soluble sugar can act as signal transduction molecules to regulate the development and adaptation to environmental challenges (Durán-Soria et al., 2020). Even though soluble sugar significantly influences the sensory quality, few studies on the sweetness of heading leaves in Chinese cabbage have yet been reported. The sweetness of vegetables is mainly related to the composition and type of soluble sugar. The biosynthesis and metabolism of soluble sugar were involved in many biological processes regulated by the expression of many genes of enzymes. INV (Invertase), SUS (sucrose synthase) and SPS (sucrose phosphate synthase) have been recognized as the essential enzymes affecting the metabolism and accumulation of soluble sugar (Nookaraju et al., 2010; Matsukura, 2016). The biosynthesis of sucrose is probably regulated by PFK6 and SUS1/SuSy1 in Chinese cabbage, and it is also regulated by SPS4, a crucial enzyme in sucrose synthesis (Li et al., 2019). During the soluble sugar biosynthesis and metabolic process of Chinese cabbage, HxK (hexokinase) and FRK (fructokinase) were involved in the further utilization of sucrose degradation products, including glucose and fructose (Moscatello et al., 2011; Hu et al., 2016).

Even though the soluble sugar and glucosinolate have the most significant influence on the flavor and quality (Wang et al., 2011; Vallone et al., 2013), their regulatory mechanism in Chinese cabbage is still unclear. Compared to white inner-leaf Chinese cabbage, yellow inner-leaf Chinese cabbage showed high glucosinolate content and low soluble sugar. Therefore, it provided an ideal experimental material to study the biosynthesis mechanism of soluble sugar and glucosinolate in Chinese cabbage. Integration of transcriptome and metabolome information offers unique insights into pathways associated with agronomy traits while identifying potential targets for genetic modification. To clarify the regulatory pathways of glucosinolate and soluble sugar in Chinese cabbage, we first investigated the differences in the accumulation of glucosinolate and soluble sugar between the yellow and the white inner-leaf Chinese cabbage, and the expression of biosynthesis-related genes of glucosinolate and soluble sugar was quantified. Then, to explain nutrient differences between the yellow and the white inner-leaf Chinese cabbage, we used conjoint transcriptome and metabolome analysis to identify different metabolites and DEGs (differentially expressed genes) in glucosinolate and soluble sugar biosynthesis. In addition, a series of physiological tests were carried out to evaluate the commercial value of the varieties. Besides, the qRT-PCR (Quantitative Real-time Polymerase Chain Reaction) was used to verify the transcriptome analysis results. Our study can provide new insights into understanding the nutrient formation of Chinese cabbage.

## Material and methods

### Plant materials and treatments

Three varieties of yellow inner-leaf Chinese cabbage, Wawahuang (WWH), Xiqing (XQ), Gaochunhuanag (GCH) and three types of white inner-leaf Chinese cabbage, Rewangzi (RWZ), Xiayangwang (XYW), Rejiangjun (RJJ) were grown and collected from an experiment field of Shandong Academy of Agricultural Sciences (Jinan city, Shandong Province) in 2021. The middle third of the edible part was cut as selected material for measuring of total soluble solids, total carotenoid, total glucosinolate, the metabolomics and RNA-Seq analysis, and qRT-PCR.

Seeds of “XQ” and “XYW” were germinated in a plastic pot (10 ×10 cm) containing soil and vermiculite at a volume ratio of 1:3 and grown under controlled conditions, 20°C ± 2, in a plant culture room in the institute of Vegetables, Shandong Academy of Agricultural Sciences. One-month-old seedlings with 5-7 leaves were selected, and the whole plants were cut up for the qRT-PCR analysis.

We used three biological replicates for one experiment, and each replicate consisted of three Chinese cabbage. Therefore, three Chinese cabbage were cut up, mixed as one sample and three biological replicates for one experiment. The samples were frozen in liquid nitrogen and stored at -80°C for further research.

### Total carotenoid content measurement

1.0 g fresh Chinese cabbage was ground in 50 ml 100% acetone and extracted in the dark at 4°C in the dark. The absorbance of the extraction solution was measured in A663, A645, and A470 (Cao et al., 2007). We calculated the total carotenoid by the following formula:


$$\text{Chlorophyll a}\;(\text{mg}/\text{L})\;=\;12.21\times {\text{A}}_{663}-2.81\times {\text{A}}_{646}$$

$$\text{Chlorophyll b}(\text{mg}/\text{L})\;=\;20.13\times {\text{A}}_{646}-5.03\times {\text{A}}_{663}$$

$$\text{Carotenoids}(\text{mg}/\text{L})\;=\;\frac{\left(1000\times {\text{A}}_{470}-3.27\times \text{Chlorophyll a}-104\times \text{Chlorophyll b}\right)}{229}$$

### Total soluble sugar content measurement

1.0 g fresh Chinese cabbage was ground and rinsed with 5 to 10 ml ddH₂O, then the extraction was filtered and boiled for 30 min, and the tube and residue were flushed to the final volume (100 ml). In the next step, we added 0.5 ml extract solution and 1.5 ml distilled water to a 25 ml glass tube with 0.5 ml anthrone-ethyl acetate and 5 ml concentrated sulfuric acid. Later, we boiled the sample for 1 min. Finally, we measured the absorbance of the reaction in A630 (Cao et al., 2007).

### Total glucosinolate content measurement

We dried 10 g of Chinese cabbage at 120°C for 15 minutes and then incubated it at 80°C for 2 days. Then, the samples were ground into powder and filtered by 100 mesh to remove the large debris. First, we added 0.1 g powder to 1.5 ml of 90% ethanol and incubated at it 70°C for 45 min. Then we filtered and washed using ddH₂O to 10 ml. Later, we added 1.0 ml solution with 2.0 ml 4 mmol/L PdCl and kept it at room temperature for 2 hours. Finally, we measured the absorbance of the reaction in A540 (Chelimoge et al., 2014).

### Metabolomics analysis of soluble sugar in Chinese cabbage

Soluble sugar content and composition were detected by MetWare (Wuhan, China, http://www.metware.cn/). An electronic mill ground the freeze-dried samples at 30 Hz for 1.5 min. 500 μL mixture with the volume ratio of methanol, isopropanol and water (3:3:2 V/V/V) was used to dissolve 20 mg sample powder and vortexed for 3 min and ultrasound for 30 min. After centrifugation at 14,000 rpm under 4°C for 3 min. The supernatant was evaporated under an N_2_ stream, and then lyophilized. The residue was used for further derivatization. A gas chromatograph and mass spectrometer (GC-MS) were used to analyse Chinese cabbage’s soluble sugar. 1.0 mL/min of pure Helium was used as a carrier gas. 1 μL gas was injected with a split ratio of 5:1. The heating program of the column oven was conducted as follows: starting at 170°C for 2 min, and then raised to 240°C at 10°C/min, the temperature increased to 280°C at 5°C/min, the temperature increased at 25°C/min to 310°C, and maintained at 310°C for 4 min. We used a selective ion monitoring mode to analyze all of the samples. The ion source and transfer line temperatures were 230°C and 240°C, respectively.

### Metabolomics analysis of glucosinolate in Chinese cabbage

Glucosinolate content and composition were detected by MetWare (Wuhan, China, http://www.metware.cn/). The sample preparation was the same as the soluble sugar measurement. 1.2 mL 70% methanol (70:30, v/v) was added into 100 mg lyophilized powder and blended. Extraction was carried out in a refrigerator at 4°C overnight. The samples were centrifuged at 12000 rpm for 10 min to remove the undissolved residue, and then were filtrated *via* SCAA-104 with 0.22 μm pore size (ANPEL, Shanghai) before being done UPLC-MS/MS analysis. Glucosinolate metabolites were measured by a UPLC-ESI-MS/MS system (UPLC, SHIMADZU Nexera X2; MS, Applied Biosystems 4500 Q TRAP) equipped with SB-C18 (1.8 µm, 2.1 mm × 100 mm). Solvent A (pure water with 0.1% formic acid) and solvent B (acetonitrile with 0.1% formic acid) were used as the UPLC mobile phase. Sample measurements were carried out with a gradient program with 0.35 mL/min flow rate and 40°C of the column oven temperature. The injection volume was 4 μL. The gradient program was started with 95% A:5% B, followed by 5% A: 95% B at 0-10 min, and at 11.1-14 min, 95% A: 5% B. The effluent was connected to an ESI-triple quadrupole-linear ion trap (QTRAP)-MS. We carried linear ion trap (LIT), and triple quadrupole (QQQ) scans on an AB4500 Q TRAP UPLC/MS/MS System equipped with an ESI Turbo Ion-Spray interface, operating in both positive and negative ion mode and controlled by the Analyst 1.6.3 software (AB Sciex). The ESI conditions were set as follows: a turbo spray ion source, 5500 V (positive ion mode)/-4500 V (negative ion mode) and 550°C; ion source gas I (GSI), gas II (GSII) and curtain gas (CUR) was set at 50, 60, and 25.0 psi, respectively; the collision-activated dissociation (CAD) was high. 10 and 100 μmol/L polypropylene glycol solutions in QQQ and LIT were conducted for Instrument tuning and mass calibration, respectively. The nitrogen was placed in the medium for the MRM experiment. Declustering potential (DP) and collision energy (CE) was done for each MRM transition.

### RNA-Seq analysis

The RNA-Seq was performed by MetWare (Wuhan, China). Total RNA was extracted from six Chinese cabbage. A total amount of 1 µg RNA per sample was used as input material for the RNA sample preparations. Sequencing libraries were generated using NEBNext^®^ UltraTM RNA Library Prep Kit for Illumina^®^ (NEB, USA). After the raw data were filtered, the sequencing error rate and the GC content distribution were checked, clean reads were obtained for subsequent analysis, and the mapped data were obtained by sequence alignment with the Chinese cabbage reference genome (http://brassicadb.cn). FPKM (fragments per kilobase of transcript per million fragments) values were used to indicate transcript or gene expression levels. The original count data were analyzed by using DESeq2 v1.22.1 software. After difference analysis, the hypothesis test probability (P-value) was corrected using the Benjamini-Hochberg method, and the FDR (the false discovery rate) was obtained. DEG screening criteria were a |log 2fold change| ≥ 1 and an FDR< 0.05. The enrichment analysis is based on the hypergeometric test using KEGG (Kyoto Encyclopedia of Genes and Genomes), and the hypergeometric distribution test is performed with the unit of the pathway. In addition, the genes showing expression values (an averaged NRPKM from three replicates) were higher than one was selected, and WGCNA (weighted gene co-expression network analysis) was performed using the WGCNA v1.69 software package.

### qRT-PCR analysis

The total RNA of six Chinese cabbage flesh frozen samples were extracted and used as a template, and a Takara Kit (PrimeScript 1st strand cDNA Synthesis Kit) was used to reverse-transcribe RNA into cDNA. Reactions were carried out on a Roche LightCycler 96 qRT-PCR detection system. The analysis of each sample was repeated three times, and the 2^−ΔCT^ method was used for quantitative data analysis. We used *Actin* as an internal reference gene. In this study, all the primers (primers synthesized by Qingdao WeiLai Biotechnology Co., Ltd.) are shown in **Supplementary Material 2**- **Supplementary Table S1**.

### Statistical analysis

KEGG annotation and enrichment analysis were used to test the statistical enrichment of the DEGs in KEGG pathways. Three biological replicates were performed in all the experiments in this study. Statistical significance (Student’s t-tests) and Pearson correlation coefficients were analyzed by using SPSS v24.0 software (SPSS Inc., Chicago, IL, USA), and a difference was considered to be statistically significant when P ≤ 0.05.

## Results

### Differences in carotenoid, soluble sugar and glucosinolate content between yellow and white inner-leaf Chinese cabbage

**Figure 1:**
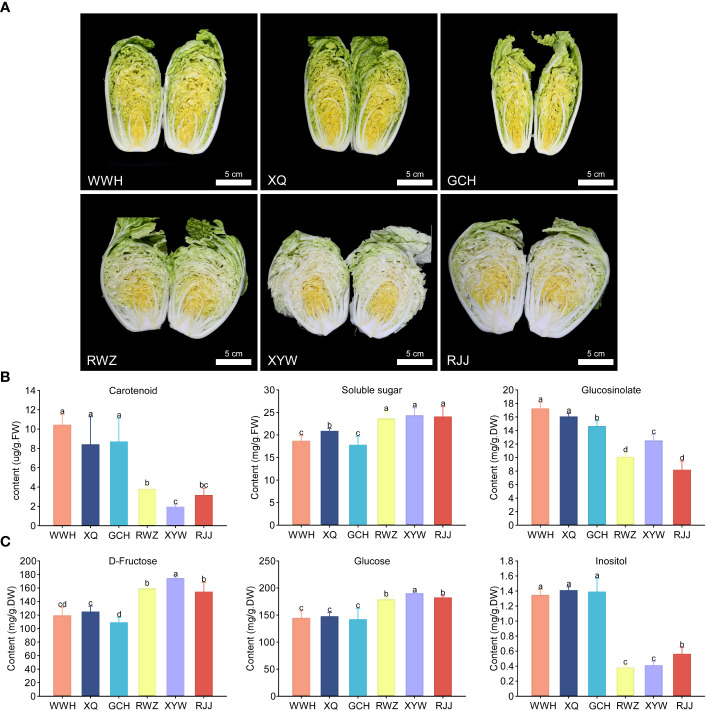
The contents of carotenoid, soluble sugar and glucosinolate in yellow and white inner-leaf Chinese cabbage. **(A)** the phenotype of yellow (WWH, XQ, GCH) and white (RWZ, XYW, RJJ) inner-leaf Chinese cabbage. Scale bars are 5 cm. **(B)** Carotenoid, Soluble sugar and Glucosinolate content. **(C)** D-Fructose, Glucose and Inositol content. Data are means ± SD, N = 3. Different letters indicate significant differences among six Chinese cabbage by t-tests (p ≤ 0.05).

Six representative Chinese cabbage cultivars with yellow or white inner-leaf color, WWH, XQ, GCH, RWZ, XYW and RJJ, were selected for this study. The inner-leaf color of WWH, XQ and GCH are yellow, while those of RWZ, XYW and RJJ are white (**Figure 1A**). The content of total carotenoid, total soluble sugar and total glucosinolate in these six cultivars of Chinese cabbage were measured (**Figure 1B**). The yellow inner-leaf Chinese cabbage observed a significantly high total carotenoid content. Similarly, the glucosinolate content in the yellow inner-leaf Chinese cabbage was generally higher than that in the white inner-leaf Chinese cabbage. Meantime, we measured soluble sugar content that could affect the taste and sweetness. We found that the rule of total soluble sugar content in yellow and white inner-leaf Chinese cabbage was opposite to carotenoid and glucosinolate content, showing a significantly high soluble sugar content in white inner-leaf Chinese cabbage.

### Qualitative and quantitative analysis of the soluble sugar and glucosinolate

To investigate the soluble sugar content and type in Chinese cabbage, we measured 13 soluble sugars using HPLC-MS/MS. As a result, there were 10 soluble sugars, including Maltose, Sucrose, Trehalose, D-Arabinose, L-Fucose, Glucose, Inositol, L-Rhamnose, and Xylitol were identified in white and yellow inner-leaf Chinese cabbage (**Supplementary Material 1**). In particular, D-fructose and glucose were the major components of soluble sugar in Chinese cabbage, accounting for more than 96% of the total soluble sugar content. Therefore, it is speculated that the significant difference between D-fructose and glucose content leads to the substantial difference in total soluble sugar content between the yellow and the white inner-leaf Chinese cabbage (p ≤ 0.05). In addition, inositol is a kind of hygienic component. However, the total content was relatively low, significantly higher in yellow inner-leaf Chinese cabbage than in white inner-leaf Chinese cabbage (**Figure 1C**).

Based on fold change ≥ 2 or ≤ 0.5 and VIP ≥ 1, the glucosinolate metabolites in the yellow and the white inner-leaf Chinese cabbage were quantitatively and qualitatively analyzed. Fifty-three glucosinolate metabolites were identified and divided into four classes, including thirty-eight aliphatic glucosinolates, nine aromatic glucosinolates, four indole glucosinolate, and one thiocyanate (**Supplementary Material 1**). Among the 53 glucosinolates, 5-Hexenyl Glucosinolate, 3-Methylamylthioglycoside, N-Hexyl glucoside, and 2-Hydroxy-4-Pentenylglucosinolate in yellow inner-leaf Chinese cabbage was generally much higher than that in white inner-leaf Chinese cabbage (**Table 1**). Furthermore, the qualitative analysis showed that all four glucosinolates belong to aliphatic glucosinolate. Therefore, we speculated that the difference in total glucosinolate content between yellow and white inner-leaf Chinese cabbage was mainly caused by the significant difference in the content of these four aliphatic glucosinolates.

**Table 1:** Compares glucosinolate content between yellow inner-leaf Chinese cabbage and white inner-leaf Chinese cabbage.

Compounds	Fold change								
	WWH/RWZ	XQ/RWZ	GCH/RWZ	WWH/XYW	XQ/XYW	GCH/XYW	WWH/RJJ	XQ/RJJ	GCH/RJJ
**5-Hexenyl Glucosinolate**	4.15	4.43	5.09	6.37	6.81	7.81	3.83	4.09	4.69
**N-Hexyl glucoside**	88500*	104000*	112000*	4.34	5.08	5.49	2.98	3.49	3.77
**3-Methylamylthioglycoside**	3.09	3.52	3.93	4.65	5.30	5.91	3.14	3.58	3.99
**2-Hydroxy-4-Pentenylglucosinolate**	2.67	2.44	2.59	2.84	2.59	2.74	3.35	3.06	3.24
**3-Hydroxy-5-(methylthio)pentyl Glucosinolate**	0.18	0.28	0.15	0.29	0.44	0.24	0.23	0.35	0.19
**3-(Methylthio)propyl glucosinolate**	0.27	0.39	0.31	0.25	0.36	0.29	0.20	0.29	0.23
**2(R)-Hydroxy-2-Phenylethyl Glucosinolate**	0.23	0.24	0.23	0.30	0.31	0.30	0.19	0.19	0.19
**5-(Methylsulfinyl)amyl glucoside**	0.17	0.24	0.14	0.29	0.42	0.23	0.18	0.26	0.15
**4-Methylthiobutyl glucosinolate (Glucoerucin)**	0.06	0.11	0.03	0.04	0.06	0.02	0.04	0.06	0.02
**5-Methylthiopentyl glucosinolate (Glucoberteroin)**	0.25	0.32	0.14	0.29	0.36	0.16	0.26	0.32	0.14
**Sulforaphane (4-methylsulphinylbutyl glucosinolate)**	0.15	0.19	0.11	0.73	0.09	0.06	0.10	0.13	0.08
**4-Methylsulfinylbutyl glucosinolate (Glucoraphanin)**	0.04	0.06	0.03	0.04	0.07	0.03	0.03	0.05	0.02
**Glucocheirolin**	0.15	0.19	0.15	0.17	0.21	0.17	0.10	0.13	0.10
**3-Phenylpropyl Glucosinolate**	0.23	0.29	0.12	0.25	0.31	0.13	0.19	0.24	0.10

*means not detected in RWZ.

### DEGs between yellow and white inner-leaf Chinese cabbage

**Figure 2:**
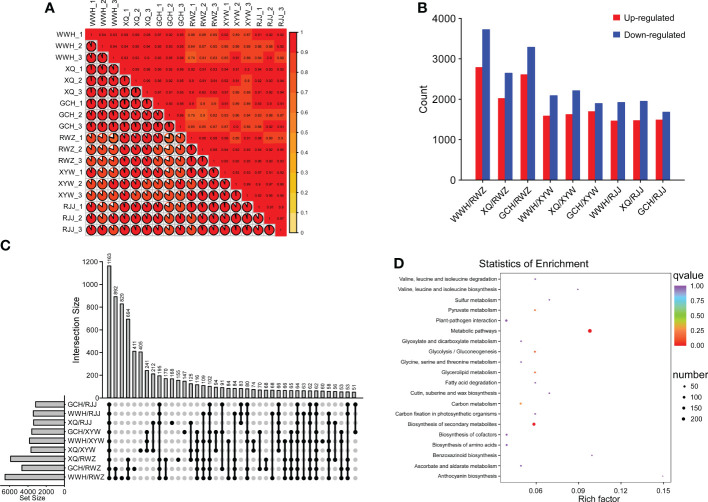
Preliminary analysis of transcriptome data. **(A)** heat map of correlation in different comparison groups. **(B)** The number of DEGs in each group. **(C)** The number of common DEGs in white and yellow inner-leaf Chinese cabbage (|log2Fold Change| ≥ 1, FDR< 0.05). **(D)** KEGG enrichment analysis of DEGs between the comparison groups (Yellow vs White inner-leaf Chinese cabbage). Each bubble in the plot represents a metabolic pathway. A larger bubble size indicates a larger impact factor. Darker bubble colors represent a higher degree of enrichment.

Based on the metabolomic analysis and the content of soluble sugar and glucosinolate, the significant differences focused on soluble sugar and glucosinolate biosynthesis pathway between yellow and white inner-leaf Chinese cabbage. Therefore, we performed RNA-Seq on yellow and white inner-leaf Chinese cabbage to study the molecular regulatory mechanisms of soluble sugar and glucosinolate biosynthesis. The 18 transcriptome samples produced 125.2 Gb Clean Data, more than 6.0 Gb per sample, with a percentage of Q30 bases above 93%. The clean reads were compared and annotated. To better elucidate the difference between yellow and white inner-leaf Chinese cabbage, the Pearson correlation coefficient (r^2^, PCC) of RNA-Seq datasets was conducted to analyze the correlation of samples (**Figure 2A**). The closer the r^2^ is to 1, the stronger the correlation between the replications of samples. The results showed a good correlation between each sample in this study was good. The PCA (principal component analysis) score plot showed that yellow inner-leaf Chinese cabbage exhibited an apparent separation from white inner-leaf Chinese cabbage, and three biological replicates of each variety were compactly gathered together (**Supplementary Material 2-Supplementary Figure 1A**), indicating that the experiment was reproducible and reliable. This comparison showed significant differences between yellow and white inner-leaf Chinese cabbage (p ≤ 0.05). In addition, 18 samples were divided into four groups in the cluster and correlation analysis on the heatmap (**Supplementary Material 2-Supplementary Figure 1B**), indicating significant differences in the contents of metabolites in yellow and white inner-leaf Chinese cabbage. These results suggested enormously different metabolite profiles in different inner-leaf color Chinese cabbage.

FPKM values were calculated for each sample to normalize the expression and further investigate the gene expression patterns. A pairwise comparison was made between yellow inner-leaf Chinese cabbage and white inner-leaf Chinese cabbage. The results showed that the most DEGs were observed in the comparison of WWH/RWZ, and the fewest DEGs were found in comparing GCH/XYW (**Figure 2B**). A total of 1163 DEGs (padj-value< 0.05, |log2Fold Change| ≥ 1), including 571 up-regulated genes and 592 down-regulated genes, were detected between yellow and white inner-leaf Chinese cabbage (**Figure 2C**). To analyze the function of common DEGs during soluble sugar and glucosinolate biosynthesis, we carried KEGG enrichment analysis between white and yellow inner-leaf Chinese cabbage (**Figure 2D**). These 1163 DEGs were enriched mainly in the metabolic pathway (ko01100) related to 203 DEGs, followed by the biosynthesis of secondary metabolites (ko01110), which underlies the soluble sugar and glucosinolate difference between white and yellow inner-leaf Chinese cabbage.

**Figure 3:**
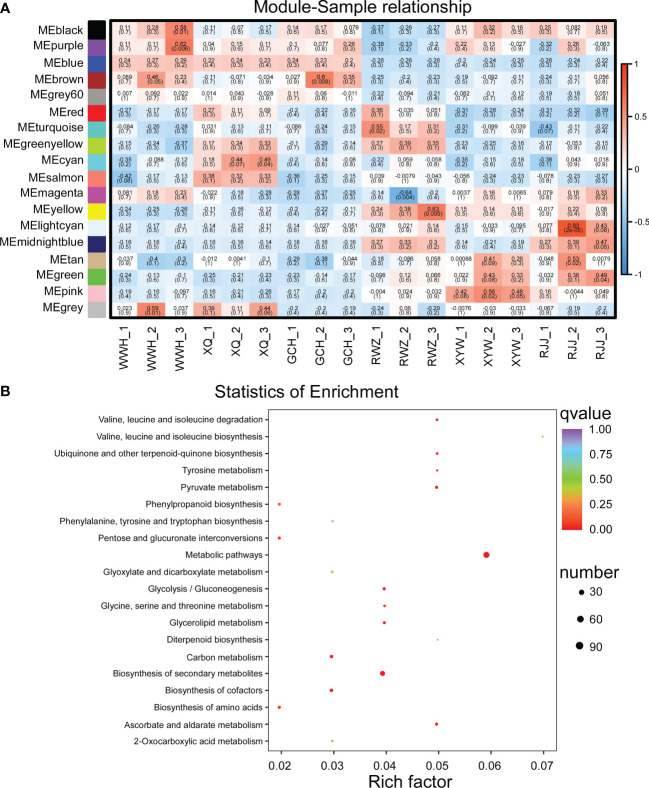
WGCNA of RNA-Seq data. **(A)** Eigen gene adjacency heatmap. **(B)** KEGG enrichment of DEGs in the blue module. Each bubble represents a metabolic pathway. A larger bubble size indicates a more considerable impact factor. The bubble colors represent the p-values of the enrichment analysis.

To gain further insight into the regulation of soluble sugar and glucosinolate biosynthesis, we carried out WGCNA to investigate the co-expression gene modules and the critical modules involved in soluble sugar and glucosinolate biosynthesis. A total of 18 co-expression modules were identified according to their expression patterns (**Figure 3A**). The correlation between the gene matrix of different modules and samples of Chinese cabbage was analyzed, and the correlation and corresponding e-value were presented in a digital form in the grid where each module and trait intersect (**Figure 3A**). According to the ‘module character’ correlation analysis, the blue module showed a significant positive correlation with all yellow inner-leaf Chinese cabbage and a negative correlation with all white inner-leaf Chinese cabbage. In addition, we carried out KEGG enrichment analyses of the blue module (**Figure 3B**). These DEGs in the blue module were enriched mainly in the metabolic pathway, followed by the biosynthesis of secondary metabolites. We found that more than 60% of DEGs between the yellow and the white inner-leaf Chinese cabbage enriched in the metabolic pathway and secondary metabolite synthesis pathway in **Figure 3** existed in the blue module, signifying blue module should be strongly correlated to the metabolism of the Chinese cabbage, including soluble sugar and glucosinolate metabolism. These results indicated that the blue module is the critical module regulating soluble sugar and glucosinolate biosynthesis in Chinese cabbage.

### Analysis of soluble sugar biosynthetic pathway in Chinese cabbage

**Figure 4:**
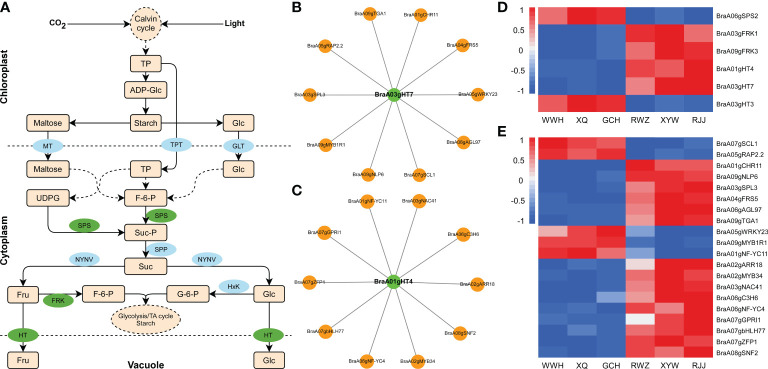
Pathway related to soluble sugar biosynthesis in Chinese cabbage. **(A)** Reconstruction of the soluble sugar biosynthetic pathway with the differentially expressed structural genes. Green ellipses represent essential genes we identified by RNA-Seq, and blue ellipses represent the genes involved in the glucosinolate biosynthesis. **(B)** Heatmap of the DEGs involved in soluble sugar biosynthetic pathway. **(C)** Correlation network of essential gene and transcription factors in the blue module. **(D)** Correlation network of structural genes and transcription factors in the green module. Green circles represent structural genes, and orange circles represent TFs. **(E)** The expression of transcription factors involved in soluble sugar biosynthesis in RNA-Seq result.

To generate the regulatory network associated with soluble sugar biosynthesis, we constructed the soluble sugar metabolic pathway in **Figure 4A**, and examined the structural genes involved in soluble sugar biosynthesis. The structural genes and transcription factors were organized into a connection network using Cytoscape software (**Figures 4B, C**). Among 1163 DEGs, six structural genes strongly correlated to the biosynthesis of soluble sugar were identified (Pearson correlation coefficients > 0.8, p-value< 0.05), including *BraA06gSPS2*, *BraA03gFRK1*, *BraA09gFRK3*, *BraA01gHT4* (hexose transporter), *BraA03gHT7*, *BraA03gHT3*. According to the analysis of gene function and RNA-Seq, *BraA01gHT4* and *BraA03gHT7* were positively correlated to the soluble sugar content, while the negative correlations were shown with *BraA03gFRK1*, *BraA09gFRK3*, *BraA06gSPS2* and *BraA03gHT3* in Chinese cabbage (**Figure 4D**). According to the soluble sugar metabolic pathway, HT mainly transported fructose and glucose from the cytoplasm to the vacuole. *BraA01gHT4* and *BraA03gHT7* encode the HT enzymes, regulating fructose and glucose metabolism*. BraA03gHT7* is in the blue module, and *BraA01gHT4* is clustered in the green module. According to weight value, the top ten transcription factors were selected from 1163 DEGs in the blue and green modules, respectively. In the blue modules, these ten transcription factors include *BraA01gCHR11* (CHROMATIN-REMODELING PROTEIN 11), *BraA05gRAP2.2* (RELATED TO AP2.2), *BraA07gSCL1* (SCARECROW-LIKE 1), *BraA09gNLP6* (NIN-LIKE PROTEIN 6), *BraA05gWRKY23*, *BraA03gSPL3* (SQUAMOSA PROMOTER BINDING PROTEIN-LIKE 3), *BraA04gFRS5* (FAR1-RELATED SEQUENCE 5), *BraA06gAGL97* (AGAMOUS-LIKE 97), *BraA09gTGA1* (TGACG SEQUENCE-SPECIFIC BINDING PROTEIN 1), and *BraA09gMYB1R1* and include *BraA01gNF-YC11* (NUCLEAR FACTOR Y, SUBUNIT C11), *BraA02gARR18* (RESPONSE REGULATOR 18), *BraA02gMYB34*, *BraA03gNAC41* (NAC DOMAIN CONTAINING PROTEIN 41), *BraA06gC3H6* (Zinc finger C-x8-C-x5-C-x3-H type family protein), *BraA06gNF-YC4*, *BraA07gGPRI1* (GBF’S PRO-RICH REGION-INTERACTING FACTOR 1), *BraA07gbHLH77* (Basic helix-loop-HELIX 77), *BraA07gZFP1* (ZINC-FINGER PROTEIN 1), and *BraA08gSNF2* (Helicase protein with RING/U-box domain-containing protein) in the green module. The structural genes and transcription factors were organized into a connection network using Cytoscape software. Interestingly, four common transcription factors, including *BraA01gCHR11*, *BraA05gRAP2.2*, *BraA07gSCL1*, and *BraA09gNLP6*, were recognized both in the biosynthesis of glucosinolate and soluble sugar in the blue module, whose expression was highly correlated with the glucosinolate and soluble sugar content (**Figures 4E**), suggested that these four transcription factors not only correspond to the regulation of glucosinolate biosynthesis but also the regulated soluble sugar biosynthesis.

### Analysis of the glucosinolate biosynthetic pathway in Chinese cabbage

**Figure 5:**
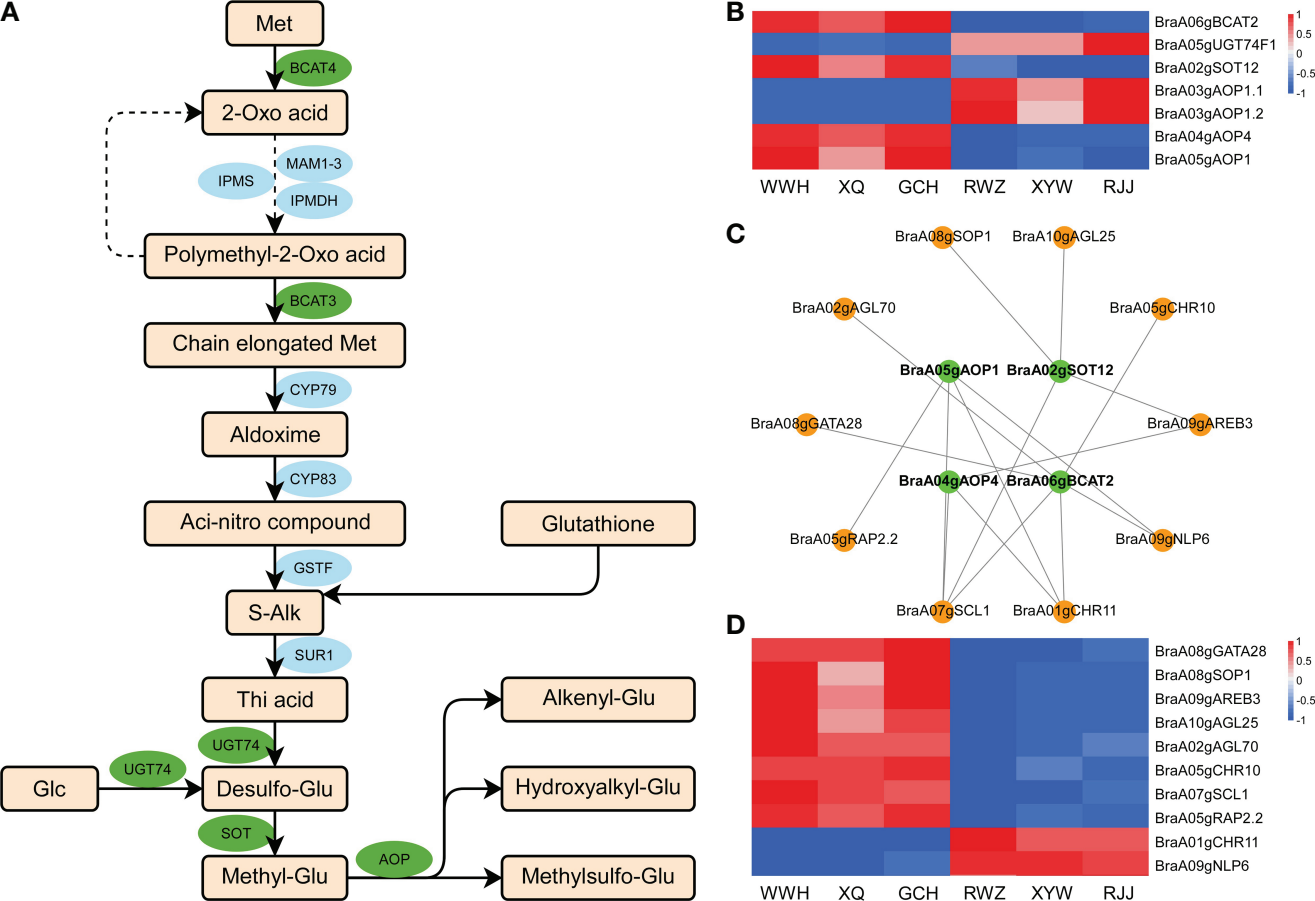
Aliphatic glucosinolate biosynthesis in Chinese cabbage. **(A)** Reconstruction of the aliphatic glucosinolate biosynthetic pathway with the differentially expressed structural genes. Green ellipses represent essential genes we identified by RNA-Seq; blue ellipses represent the genes involved in the glucosinolate biosynthesis. **(B)** Heatmap of the DEGs involved in aliphatic glucosinolate biosynthesis. **(C)** Correlation network of crucial genes involved in aliphatic glucosinolate biosynthesis in yellow and white inner-leaf Chinese cabbage. Green circles represent structural genes; orange circles represent TFs. **(D)** The expression of transcription factors involved in glucosinolate biosynthesis in RNA-Seq result.

To further investigate the regulation mechanism underlying glucosinolate between yellow and white inner-leaf Chinese cabbage, we analyzed the aliphatic glucosinolate biosynthetic pathway to perform the critical genes in the metabolism of yellow and white inner-leaf Chinese cabbage. The biosynthesis of aliphatic glucosinolate is derived from Met. *BCAT*, *MAM*, *CYP79/CYP83*, *AOP*, *SOT*, and *UGT74* regulate aliphatic glucosinolate biosynthesis (**Figure 5A**). Among 1163 DEGs, seven structural genes involved in aliphatic glucosinolate biosynthesis, including *BraA05gUGT74F1*, *BraA03gAOP1.2*, *BraA03gAOP1.1*, *BraA06gBCAT2*, *BraA02gSOT12*, *BraA04gAOP4*, and *BraA05gAOP1* were identified (**Figure 5B**). Correlation analysis was conducted between the expression of these structural genes and aliphatic glucosinolates, significantly different in yellow and white inner-leaf Chinese cabbage (PCC were calculated using the cor function in R). *BraA05gAOP1*, *BraA02gSOT12*, *BraA06gBCAT2*, and *BraA04gAOP4* located in the blue module were positively correlated to the glucosinolate content in yellow inner-leaf Chinese cabbage (PPC>0.8, p-value<0.05). While *BraA05gUGT74F1*, *BraA03gAOP1.2*, and *BraA03gAOP1.1* were negatively correlated to the glucosinolate content in yellow inner-leaf Chinese cabbage.

The top ten transcription factors in the blue modules were selected based on weight values from the 1163 DEGs. The visualization network between structural genes and transcription factors (Cytoscape software) was shown in **Figure 5C**. Among these ten transcriptions factors, eight of them, including *BraA08gGATA28* (BraA08g002520.3C)*, BraA08gSOP1* (SUPPRESSOR OF PAS2 1)*, BraA09gAREB3* (ABA-RESPONSIVE ELEMENT BINDING PROTEIN 3)*, BraA10gAGL25, BraA02gAGL70, BraA05gCHR10, BraA05gRAP2.2*, and *BraA07gSCL1* were up-regulated (**Figure 5D**). At the same time, *BraA01gCHR11* and *BraA09gNLP6* were down-regulated in yellow inner-leaf Chinese cabbage, suggesting positive and negative regulation of glucosinolate biosynthesis, respectively (**Figure 5D**). These results indicated that these ten transcription factors correspond to the putative regulators controlling glucosinolate biosynthesis in Chinese cabbage.

### Confirmation of the transcriptome data using qRT-PCR

**Figure 6:**
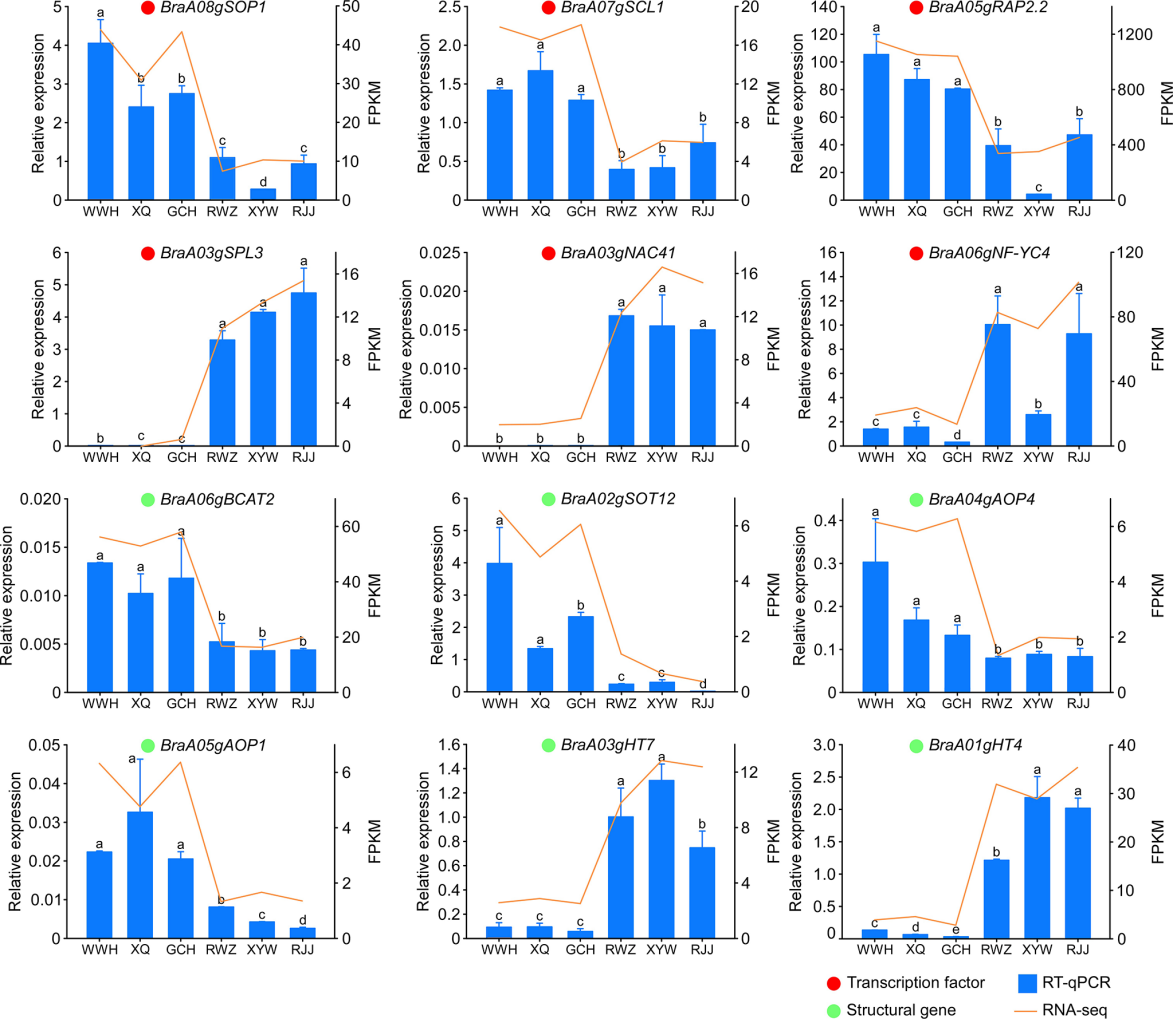
qRT- PCR verification of expression levels of key genes identified by RNA sequencing. The relative expression levels analyzed by qRT-PCR and calculated by 2^-△Ct^ and bars with different lowercase letters are significantly different (P ≤ 0.05). The red circle represents transcription factors, and the green circle represents structural genes.

To validate the transcriptome data, the expression pattern of the structural genes which we identified related to the biosynthesis of soluble sugar (*BraA03gHT7* and *BraA01gHT4*) and glucosinolate (*BraA06gBCAT2, BraA02gSOT12, BraA04gAOP4*, and *BraA05gAOP1*) was investigated in the yellow and the white inner-leaf Chinese cabbage by using the qRT-PCR (**Figure 6**). The relative expression of these structural genes agrees with the RNA-seq results, indicating consistency in the RNA-seq data and the qRT-PCR results. In addition, six transcription factors, including *BraA03gNAC41, BraA03gSPL3, BraA05gRAP2.2, BraA06gNF-YC4, BraA07gSCL1*, and *BraA08gSOP1* were selected randomly from the candidate genes for the regulation of glucosinolate and soluble sugar biosynthesis, and their expression was analyzed using qRT-PCR to validate the transcriptome data sets from RNA-Seq. The results showed that the relative expression trends of these transcription factors are in close agreement with the corresponding relative transcript abundances, validating the reproducibility and credibility of the transcriptome data in this study.

### Expression analysis of genes related to the biosynthesis of soluble sugar and glucosinolate

**Figure 7:**
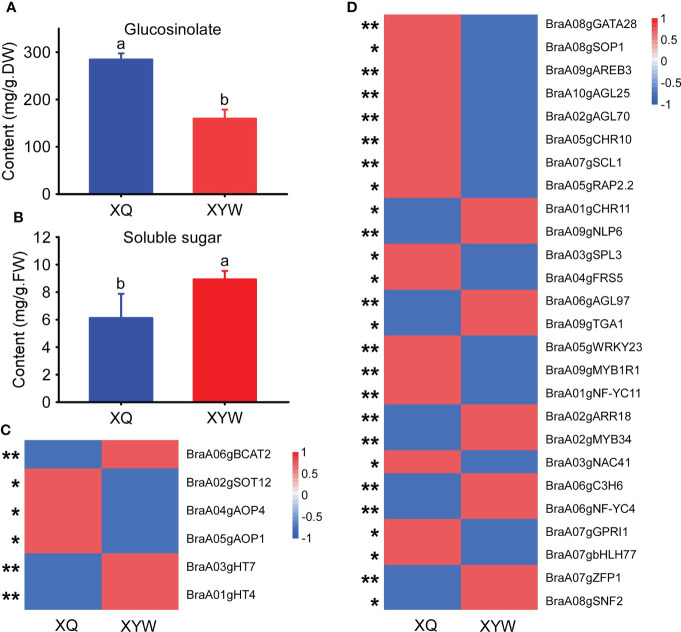
Expression analysis of key genes related to soluble sugar and glucosinolate biosynthesis in yellow and white inner-leaf Chinese cabbage. **(A)** Glucosinolate content at the seedling stage. **(B)** Soluble sugar content at the seedling stage. Data are means ± SD, N = 3. Lowercase letters indicate that there are significant differences between ‘XQ’ and ‘XYW’ (P ≤ 0.05). **(C)** Relative expression of structural genes involved in glucosinolate and soluble sugar biosynthesis at the seedling stage. **(D)** The expression of transcription factors involved in glucosinolate and soluble sugar biosynthesis at the seedling stage. Asterisks indicate statistical significance using Student’s t-test: *,P ≤ 0.05, **,P ≤ 0.01.

The seedling stage of yellow inner-leaf (XQ) and white inner-leaf (XYW) Chinese cabbage were used to verify the above results and confirm the molecular mechanism of Chinese cabbage soluble sugar and glucosinolate biosynthesis. We measured the total soluble sugar content and total glucosinolate content of the seeding stage in ‘XQ’ and ‘XYW’ (**Figures 7A, B**). The results showed a high glucosinolate in yellow inner-leaf Chinese cabbage. And, a significant high soluble sugar content is observed in white inner-leaf Chinese cabbage, which is consistent to with their content at the heading stage.

The expression patterns of all structural genes and ten transcription factors regulating the biosynthesis of glucosinolate and soluble sugar were analyzed using the qRT-PCR in the seedlings of ‘XQ’ and ‘XYW’ (**Figures 7C, D**). The relative expression of four structural genes regulating glucosinolate biosynthesis was significantly higher in ‘XQ’ than in ‘XYW’. *BraA05gAOP1, BraA02gSOT12*, and *BraA04gAOP4* as the positive regulators of glucosinolate biosynthesis. It agrees with the glucosinolate content (**Figure 7A**) and the RNA-Seq data (**Figure 7C**). While the expression of the negative regulator of *BraA06gBCAT2* is inconsistent with the glucosinolate content.

Meanwhile, the relative expression level of two structural genes, *BraA01gHT4* and *BraA03gHT7*, confirmed the soluble sugar content, with the significantly high expression level in white inner-leaf Chinese cabbage. The verification of ten transcription factors correlated to *BraA01gHT4* and *BraA03gHT7* showed that *BraA03gSPL3* and *BraA04gFRS5* in the blue module are inconsistent RNA-Seq results with higher expression in white inner-leaf Chinese cabbage in RNA-Seq data but lower expression at seedling stage of white inner-leaf Chinese cabbage (**Figure 7D**). It agrees with the soluble sugar content at the heading and seedling stages. In the green module, the expression of *BraA03gNAC41*, *BraA07gGPRI1*, and *BraA07gbHLH77* were high in white inner-leaf Chinese cabbage. It is contrary to the soluble sugar content and RNA-Seq at the seedling stage.

In addition, the four common transcription factors regulating glucosinolate and soluble sugar biosynthesis, *BraA07gSCL1*, *BraA05gRAP2.2*, *BraA01gCHR11*, *BraA09gNLP6* exhibited a consistent result to RNA-Seq and glucosinolate content. However, the RNA-Seq results and the expression pattern of *BraA07gSCL1* and *BraA05gRAP2.2* showed a contrary trend to the soluble sugar content.

## Discussion

### Quantitative and qualitative analysis of soluble sugar and glucosinolate in Chinese cabbage

Flavor and leaf color are two essential quality indices of Chinese cabbage (Huang et al., 2016). The content of glucosinolate and soluble sugar determines the flavor of Chinese cabbage. In a previous study, the total glucosinolate content in Chinese cabbage was much lower than that in broccoli, kale and other cruciferous vegetables (Bhandari et al., 2015). The metabolic pathways and regulators governing the biosynthesis of soluble sugar and glucosinolate were elucidated to improve the quality of Chinese cabbage by increasing the soluble sugar and glucosinolate content. After pairwise comparison, a higher content of carotenoid and glucosinolate was found in yellow inner-leaf Chinese cabbage (**Figure 1B**). The qualitative and quantitative analysis showed that aliphatic glucosinolate was the primary type of glucosinolate in Chinese cabbage (**Supplementary Material 1**). It was consistent with the report that the content of aliphatic glucosinolate was higher than other kinds of glucosinolate and positively correlated with the carotenoid in Chinese cabbage (Baek et al., 2016). The content and type of soluble sugar are essential factors that affect the flavor and sweetness of Chinese cabbage. In our study, the soluble sugar content in white inner-leaf Chinese cabbage was generally higher than that of yellow inner-leaf Chinese cabbage. D-fructose and glucose were the main ingredients of soluble sugar in Chinese cabbage. Sucrose, fructose and glucose show significant differences in sweetness; fructose’s sweetness is 1.73 times that of sucrose and 2.34 times that of glucose (Pangborn, 1963). This result signified that fructose and glucose could be mainly responsible for the high sweetness of white inner-leaf Chinese cabbage. Although inositol was detected in both yellow inner-leaf and white inner-leaf Chinese cabbage, the content in yellow inner-leaf Chinese cabbages had a much higher range than in white inner-leaf Chinese cabbage. Inositol is not only involved in plant sugar transport and resistance but also inhibits the growth of tumor cells while also having the same function as vitamin B1 (Loewus and Loewus, 1983). This also further explains the traits of the higher nutritional value of yellow inner-leaf Chinese cabbage.

### Molecular mechanism of soluble sugar biosynthesis and accumulation in Chinese cabbage

Elucidating the metabolic pathways and regulators governing the biosynthesis and accumulation of soluble sugar is essential to provide new leads for improving Chinese cabbage quality. Fructose and glucose biosynthesis and metabolism are regulated by many essential structural genes, for instance, *SUS*, *INV*, *HxK*, *SPS*, and *FRK* (Rosa et al., 2009; Liu et al., 2020; Cheng et al., 2022). In our study, two critical structural genes encoding HT were identified by the conjugation of metabolomic and transcriptomic analyses. HT is a class of transporter proteins which directly regulates hexose content by transporting the hexose into a vacuole from the plasma membrane. As reported, the high HT activity transfers hexose into parenchyma cells against the concentration gradient and promotes sugar accumulation in fruit (Li et al., 2018). In apples with overexpression of *HT*, fructose and glucose content were significantly increased, while the sucrose content was decreased (Wang, 2020). And the *LeHT1* and *LeHT2* exhibited transport properties consistent with a high-affinity glucose/H^+^ and low-affinity fructose/H^+^ symporters (McCurdy et al., 2010) in tomatoes. Moreover, the decrease of LeHT1 caused a decline in hexoses concentrations, including glucose, fructose, and mannose (McCurdy et al., 2010). According to metabolic data analysis, fructose and glucose were the main component of soluble sugar in Chinese cabbage. Therefore, we suspected that HT probably is a key enzyme regulating the soluble sugar content in Chinese cabbage and also leads to the difference in soluble sugar between the yellow and the white inner-leaf Chinese cabbage.

Furthermore, transcription factors are the key factors in the biosynthesis of soluble sugar in Chinese cabbage. So far, *MYB*, *WARKY*, and *bZIP* have been confirmed to regulate soluble sugar biosynthesis. It has been reported that CmMYB113 could interact with CmSPS1 and CmACO1, regulating ethylene-dependent sucrose accumulation (Gao et al., 2021). *AtbZIP1* was vital in sugar-mediated gene expression and maintained sucrose homeostasis in plants (Wiese et al., 2005). Sucrose-specific signaling pathways showed to be responsible for the repression of ATB2/AtbZIP (Wiese et al., 2005). The expression of *WRKY42* is also closely related to the sucrose content in Pomegranate (Feng et al., 2022). In our study, we identified the critical transcription factors modulating the transcription of the vital structural genes, such as *WRKY*, *bZIP*, *GRAS*, *MYB*, *bHLH*, *RWP-RK*, *AP2/ERF-ERF*, and *B3*. Among them, transcription factors/transcription regulation factors *BraA07gSCL1* (GRAS), *BraA01gCHR11* (SNF2), *BraA05gRAP2.2* (AP2/ERF-ERF), and BraA09gNLP6 (RWP-RK) are involved in the regulation of both glucosinolate and soluble sugar biosynthesis. Whereas their expression levels of them at the seedling stage suggested that the *BraA09gNLP6* and *BraA01gCHR11* are not the critical transcription factors regulating soluble sugar biosynthesis in Chinese cabbage.

### Molecular mechanism of glucosinolate in Chinese cabbage

In previous studies, the biosynthesis of aliphatic glucosinolate goes through the chain elongation of selected precursor amino acids, the formation of core glucosinolates structure and the secondary modification of the amino acid side chains in three steps (Sønderby et al., 2010). The vital roles of *BCAT*, *MAM*, *CYP79/CYP83*, *AOP*, *SOT*, and *UGT74* in this process have been reported (Sanchez-Pujante et al., 2017). In previous study, the *AOP* is probably the principal regulator of aliphatic glucosinolate accumulation in the biosynthetic pathway (Kliebenstein et al., 2001; Wang et al., 2021), consistent with our results. BCAT, as an aminotransferase enzyme, can act on branched-chain amino acids to regulate sulfur amino acid metabolism (Klein et al., 2006). The function of *AtSOT16*, *AtSOT17* and *AtSOT18* sulfonated desulphoglucosinolates in the last step of glucosinolate biosynthesis also were confirmed (Hashiguchi et al., 2014). In addition, *AtSOT12* was shown to be sulphurated brassinosteroids and flavanone (Marsolais et al., 2006; Gigolashvili et al., 2007). Even though these structural genes of glucosinolate biosynthesis have been studied a lot, previous studies mainly focused on Arabidopsis, and the relevant studies on Chinese cabbage are still lacking.

In our study, at the heading stage, the expression level of structural genes, *BraA06gBCAT2*, *BraA02gSOT12*, *BraA05gAOP1*, and *BraA04gAOP4* was significantly higher in the yellow inner-leaf Chinese cabbage than that in the white inner-leaf Chinese cabbage (**Figure 7**). Nevertheless, the expression of four structural genes was significantly higher. Therefore, considering the higher expression of *BraA06gBCAT2* in white inner-leaf Chinese cabbage at the seedling stage, we proposed that *BraA06gBCAT2* might not be an essential gene regulating glucosinolates accumulation at the seedling stage.

In past reports, MYB28, MYB29, and MYB76 participated in aliphatic glucosinolate biosynthesis by regulating *MAM1*, *MAM3*, *CYP79F1*, *CYP79F2*, *CYP83A1*, *AtST5b* and *AtST5c* (Zang et al., 2008). The gain of *MYB28* function increased the content of glucosinolate while decreasing in HAG1 RNAi knock-down mutants (Schön et al., 2013). Previous studies proved that bHLH could form protein complexes with MYB and allow the coordinated and rapid regulation of glucosinolate genes in cruciferous plants (Mitreiter and Gigolashvili, 2021). In addition, WRKY18 and WRKY40 are synergistic with CYP81F2 to negatively regulate the biosynthesis of indole glucosinolate (Schweizer et al., 2013). The crucial role of the MYC-MYB interaction system has been reported in the regulation of glucosinolate biosynthesis (Zang et al., 2021). To validate the previous studies, our report can also be used to verify the regulation of transcription factors for glucosinolate accumulation in Chinese cabbage. Our dataset showed that *bZIP* (Wiese et al., 2005), *SNF2*, *GRAS*, *MYB* (Zang et al., 2021), *bHLH* (Mitreiter and Gigolashvili, 2021) et al. play an essential role in regulating structural genes of glucosinolate biosynthesis. Of all the ten transcription factors, *BraA07gSCL1* belonged to the *GRAS* transcription factor family and was noted for its strong relationship with all four structural genes. The critical role of *SCL* on plant growth and development, signal transduction, disease resistance and stress resistance has been reported (Chen et al., 2020; Wang et al., 2020; Zang et al., 2021), while a lack of direct evidence on glucosinolate accumulation require the further research and verification.

## Data availability statement

The datasets presented in this study can be found in online repositories. The names of the repository/repositories and accession number(s) can be found below: https://www.ncbi.nlm.nih.gov/, PRJNA867427.

## Author contributions

FW and JG supervised and conceived this project. LW wrote the paper. SZ and FW revised the paper. LW and SZ performed the transcript analysis and metabolic profiling. LW carried out qRT-PCR. JL, YZ, CL, LH, and HL performed sampling and preprocessing for transcriptome and metabolism analyses.

## Funding

This research was funded by the Taishan Scholars Program of Shandong Province, China (tsqn201909167); the National Natural Science Foundation, China (32172591); the Modern Agricultural Industrial Technology System Funding of Shandong Province, China (SDAIT-05); the Agricultural Science and Technology Innovation Project of SAAS, China (CXGC2022D01); the Key R&D Program of Shandong Province, China (2019GHZ014); the Shandong Upgraded Project of “Bohai Granary” Science and Technology Demonstration Engineering (2019BHLC005); the China Agriculture Research System (CARS-23-G13); the Agricultural Science and Technology Innovation Project of SAAS (CXGC2022E08); the Jinan Agricultural Application Technology Innovation Program (202009).

## Acknowledgments

Great appreciation is given to the editor and reviewers’ critical comments on the improvement of the manuscript.

## Conflict of interest

The authors declare that the research was conducted in the absence of any commercial or financial relationships that could be construed as a potential conflict of interest.

## Publisher’s note

All claims expressed in this article are solely those of the authors and do not necessarily represent those of their affiliated organizations, or those of the publisher, the editors and the reviewers. Any product that may be evaluated in this article, or claim that may be made by its manufacturer, is not guaranteed or endorsed by the publisher.

## References

[B1] Baek S. A. Jung Y. H. Lim S. H. Park S. U. Kim J. K. (2016). Metabolic profiling in Chinese cabbage (Brassica rapa l. subsp. pekinensis) cultivars reveals that glucosinolate content is correlated with carotenoid content. J. Agric. Food Chem. 64, 4426–4434. doi: 10.1021/acs.jafc.6b01323 27172980

[B2] Bhandari S. R. Jo J. S. Lee J. G. (2015). Comparison of glucosinolate profiles in different tissues of nine brassica crops. Molecules 20, 15827–15841. doi: 10.3390/molecules200915827 26334264 PMC6331803

[B3] Bischoff K. L. (2021). Chapter 53 -Glucosinolates. In Gupta R. C. Nutraceuticals(Boston: Academic Press), 551–4.

[B4] Cao J. K. Jiang W. B. Zhao Y. M. (2007). Physiological and biochemical guidance of fruits and vegetables after harvest. (Beijing: China Light Industry Press).

[B5] Cao W. Wang P. Yang L. Fang Z. Zhang Y. Zhuang M.. (2021). Carotenoid biosynthetic genes in cabbage: Genome-wide identification, evolution, and expression analysis. Genes (Basel) 12 (12), 2027–40. doi: 10.3390/genes12122027 34946976 PMC8701174

[B6] Chelimoge Yuan Q. Yang R. J. Cao X. L. Zhu S. H. (2014). 'Screening of conditions for determination of glucosinolates in rapeseed by PdCl colorimetry. Heilongjiang Anim. Sci. Vet. Med. 11, 216–217. doi: 10.13881/j.cnki.hljxmsy.2014.0450

[B7] Chen Q. J. Deng B. H. Gao J. Zhao Z. Y. Chen Z. L. Song S. R.. (2020). A miRNA-encoded small peptide, vvi-miPEP171d1, regulates adventitious root formation. Plant Physiol. 183, 656–670. doi: 10.1104/pp.20.00197 32241877 PMC7271809

[B8] Cheng H. Kong W. Tang T. Ren K. Zhang K. Wei H.. (2022). Identification of key gene networks controlling soluble sugar and organic acid metabolism during oriental melon fruit development by integrated analysis of metabolic and transcriptomic analyses. Front. Plant Sci. 13, 830517. doi: 10.3389/fpls.2022.830517 35646021 PMC9135470

[B9] Chhajed S. Mostafa I. He Y. Abou-Hashem M. El-Domiaty M. Chen S. (2020). Glucosinolate biosynthesis and the glucosinolate–myrosinase system in plant defense. Agronomy 10, 1786–1810. doi: 10.3390/agronomy10111786

[B10] Das B. (2021). Glucosinolate biosynthesis: role of MAM synthase and its perspectives. Biosci. Rep. 41, BSR20211634. doi: 10.1042/BSR20211634 34545928 PMC8490860

[B11] Durán-Soria S. Pott D. M. Osorio S. Vallarino J. G. (2020). Sugar signaling during fruit ripening. Front. Plant Sci. 11, 564917. doi: 10.3389/fpls.2020.564917 32983216 PMC7485278

[B12] Feng L. Wang C. Yang X. Jiao Q. Yin. Y. (2022). Transcriptomics and metabolomics analyses identified key genes associated with sugar and acid metabolism in sweet and sour pomegranate cultivars during the developmental period. Plant Physiol. Biochem. 181, 12–22. doi: 10.1016/j.plaphy.2022.04.007 35421745

[B13] Gao G. Duan X. Jiang H. Yang F. Qi. H. (2021). CmMYB113 regulates ethylene-dependent sucrose accumulation in postharvest climacteric melon fruit. Postharvest Biol. Technol. 181, 111682. doi: 10.1016/j.postharvbio.2021.111682

[B14] Gigolashvili T. Yatusevich R. Berger B. Muller C. Flugge U. I. (2007). The R2R3-MYB transcription factor HAG1/MYB28 is a regulator of methionine-derived glucosinolate biosynthesis in arabidopsis thaliana. Plant J. 51, 247–261. doi: 10.1111/j.1365-313X.2007.03133.x 17521412

[B15] Guo R. Huang Z. Deng Y. Chen X. XuHan X. Lai. Z. (2016). Comparative transcriptome analyses reveal a special glucosinolate metabolism mechanism in brassica alboglabra sprouts. Front. Plant Sci. 7. doi: 10.3389/fpls.2016.01497 PMC5047911 27757119

[B16] Harun S. Abdullah-Zawawi M. R. Goh H. H. Mohamed-Hussein Z. A. (2020). A comprehensive gene inventory for glucosinolate biosynthetic pathway in arabidopsis thaliana. J. Agric. Food Chem. 68 (28), 7281–7297. doi: 10.1021/acs.jafc.0c01916 32551569

[B17] Hashiguchi T. Sakakibara Y. Shimohira T. Kurogi K. Yamasaki M. Nishiyama K.. (2014). Identification of a novel flavonoid glycoside sulfotransferase in arabidopsis thaliana. J. Biochem. 155, 91–97. doi: 10.1093/jb/mvt102 24202284 PMC3905610

[B18] Higdon J. V. Delage B. Williams D. E. Dashwood R. H. (2007). Cruciferous vegetables and human cancer risk: Epidemiologic evidence and mechanistic basis. Pharmacol. Res. 55, 224–236. doi: 10.1016/j.phrs.2007.01.009 17317210 PMC2737735

[B19] Huang S. Liu Z. Li D. Yao R. Hou L. Li X.. (2016). Physiological characterization and comparative transcriptome analysis of a slow-growing reduced-thylakoid mutant of Chinese cabbage (Brassica campestris ssp. pekinensis). Front. Plant Sci. 7, 3. doi: 10.3389/fpls.2016.00003 26858733 PMC4726769

[B20] Hu J. H. Chen B. Y. Zhao J. Zhang F. Xie T. Xu K.. (2022). Genomic selection and genetic architecture of agronomic traits during modern rapeseed breeding. Nat. Genet. 54, 694–704. doi: 10.1038/s41588-022-01055-6 35484301

[B21] Hu L. Wu G. Hao C. Yu H. Tan L. (2016). Transcriptome and selected metabolite analyses reveal points of sugar metabolism in jackfruit (Artocarpus heterophyllus lam.). Plant Sci. 248, 45–56. doi: 10.1016/j.plantsci.2016.04.009 27181946

[B22] Ishida M. Hara M. Fukino N. Kakizaki T. Morimitsu Y. (2014). Glucosinolate metabolism, functionality and breeding for the improvement of brassicaceae vegetables. Breed Sci. 64, 48–59. doi: 10.1270/jsbbs.64.48 24987290 PMC4031110

[B23] Jo J. S. Bhandari S. R. Kang G. H. Shin Y. K. Lee. J. G. (2022). Selection of broccoli (Brassica oleracea var. italica) on composition and content of glucosinolates and hydrolysates. Sci. Hortic. 298, 110984. doi: 10.1016/j.scienta.2022.110984

[B24] Kang K. B. Jayakodi M. Lee Y. S. Nguyen V. B. Park H. S. Koo H. J.. (2018). Identification of candidate UDP-glycosyltransferases involved in protopanaxadiol-type ginsenoside biosynthesis in panax ginseng. Sci. Rep. 8, 11744. doi: 10.1038/s41598-018-30262-7 30082711 PMC6078999

[B25] Klein M. Papenbrock J. (2008). “Sulfotransferases and their role in glucosinolate biosynthesis,” in Sulfur assimilation and abiotic stress in plants. Eds. Khan N. A. Singh S. Umar S. (Berlin, Heidelberg: Springer Berlin Heidelberg).

[B26] Klein M. Reichelt M. Gershenzon J. Papenbrock. J. (2006). The three desulfoglucosinolate sulfotransferase proteins in arabidopsis have different substrate specificities and are differentially expressed. FEBS J. 273, 122–136. doi: 10.1111/j.1742-4658.2005.05048.x 16367753

[B27] Kliebenstein D. J. Gershenzon J. Mitchell-Olds T. (2001). Comparative quantitative trait loci mapping of aliphatic, indolic and benzylic glucosinolate production in arabidopsis thaliana leaves and seeds. Genetics 159, 359–370. doi: 10.1093/genetics/159.1.359 11560911 PMC1461795

[B28] Ku K. M. Becker T. M. Juvik J. A. (2016). Transcriptome and metabolome analyses of glucosinolates in two broccoli cultivars following jasmonate treatment for the induction of glucosinolate defense to trichoplusia ni (Hübner). Int. J. Mol. Sci. 17 (7), 1135–52. doi: 10.3390/ijms17071135 27428958 PMC4964508

[B29] Liao Y. C. (2011). 'Analysis of Structure and Content of Glucosinolates and QTL Location in Brassica RaPa (Chongqing: Southwese University).

[B30] Li Y. Fan Y. Jiao Y. Wu J. Zhang Z. Yu X.. (2019). Transcriptome profiling of yellow leafy head development during the heading stage in Chinese cabbage (Brassica rapa subsp. pekinensis). Physiol. Plant. 165, 800–813. doi: 10.1111/ppl.12784 29900559

[B31] Li M. Li P. Ma F. Dandekar A. M. Cheng L. (2018). Sugar metabolism and accumulation in the fruit of transgenic apple trees with decreased sorbitol synthesis. Hortic. Res. 5, 60. doi: 10.1038/s41438-018-0064-8 30510767 PMC6269491

[B32] Liu Q. Li J. Liu. W. (2020). Sugar accumulation and characterization of metabolizing enzyme genes in leafy head of Chinese cabbage (Brassica campestris l. ssp. pekinensis). Hortic. Environ. Biotechnol. 62, 17–29. doi: 10.1007/s13580-020-00294-y

[B33] Loewus F. A. Loewus M. W. (1983). Myo-inositol: Its biosynthesis and metabolism. Annual Review of Plant Physiology 34, 137–161.

[B34] Marsolais F. Boyd J. Paredes Y. Schinas A. M. Garcia M. Elzein S.. (2006). Molecular and biochemical characterization of two brassinosteroid sulfotransferases from arabidopsis, AtST4a (At2g14920) and AtST1 (At2g03760). Planta 225 (5), 1233–1244. doi: 10.1007/s00425-006-0413-y 17039368

[B35] Matsukura C. (2016). “Sugar accumulation in tomato fruit and its modification using molecular breeding techniques,” in Functional genomics and biotechnology in solanaceae and cucurbitaceae crops. Eds. Ezura H. Ariizumi T. Garcia-Mas J. Rose J. (Berlin, Heidelberg: Springer Berlin Heidelberg).

[B36] McCurdy D. W. Dibley S. Cahyanegara R. Martin A. Patrick J. W. (2010). Functional characterization and RNAi-mediated suppression reveals roles for hexose transporters in sugar accumulation by tomato fruit. Mol. Plant 3, 1049–1063. doi: 10.1093/mp/ssq050 20833733

[B37] Mitreiter S. Gigolashvili T. (2021). 'Regulation of glucosinolate biosynthesis. J. Exp. Bot. 72, 70–91. doi: 10.1093/jxb/eraa479 33313802

[B38] Moscatello S. Famiani F. Proietti S. Farinelli D. Battistelli. A. (2011). Sucrose synthase dominates carbohydrate metabolism and relative growth rate in growing kiwifruit (Actinidia deliciosa, cv Hayward). Sci. Hortic. 128, 197–205. doi: 10.1016/j.scienta.2011.01.013

[B39] Nookaraju A. Upadhyaya C. Pandey S. Young K. Hong S. Park S.. (2010). Molecular approaches for enhancing sweetness in fruits and vegetables. Sci. Hortic. 127, 1–15. doi: 10.1016/j.scienta.2010.09.014

[B40] Pangborn R. M. (1963). Relative taste intensities of selected sugars and organic acids. J. Food Sci. 28, 726–733. doi: 10.1111/j.1365-2621.1963.tb01680.x

[B41] Rosa M. Prado C. Podazza G. Interdonato R. Gonzalez J. A. Hilal M.. (2009). Soluble sugars–metabolism, sensing and abiotic stress: a complex network in the life of plants. Plant Signaling Behav. 4, 388–393. doi: 10.4161/psb.4.5.8294 PMC2676748 19816104

[B42] Sønderby I. E. Geu-Flores F. Halkier. B. A. (2010). Biosynthesis of glucosinolates – gene discovery and beyond. Trends Plant Sci. 15, 283–290. doi: 10.1016/j.tplants.2010.02.005 20303821

[B43] Sanchez-Pujante P. J. Borja-Martinez M. Pedreno M. A. Almagro L. (2017). Biosynthesis and bioactivity of glucosinolates and their production in plant *in vitro* cultures. Planta 246, 19–32. doi: 10.1007/s00425-017-2705-9 28492986

[B44] Schön M. Töller A. Diezel C. Roth C. Westphal L. Wiermer M.. (2013). Analyses of wrky18 wrky40 plants reveal critical roles of SA/EDS1 signaling and indole-glucosinolate biosynthesis for golovinomyces orontii resistance and a loss-of resistance towards pseudomonas syringae pv. tomato AvrRPS4. Mol. Plant Microbe Interact. 26, 758–767. doi: 10.1094/MPMI-11-12-0265-R 23617415

[B45] Schweizer F. Fernández-Calvo P. Zander M. Diez-Diaz M. Fonseca S. Glauser G.. (2013). Arabidopsis basic helix-loop-helix transcription factors MYC2, MYC3, and MYC4 regulate glucosinolate biosynthesis, insect performance, and feeding behavior. Plant Cell. 25, 3117–3132. doi: 10.1105/tpc.113.115139 23943862 PMC3784603

[B46] Shen J. Zou Z. Zhang X. Zhou L. Wang Y. Fang W.. (2018). Metabolic analyses reveal different mechanisms of leaf color change in two purple-leaf tea plant (Camellia sinensis l.) cultivars. Hortic. Res. 5, 7. doi: 10.1038/s41438-017-0010-1 29423237 PMC5802758

[B47] Shim J. Y. Kim D. G. Park J. T. Kandpal L. Hong S. J. Cho B. K.. (2016). Physicochemical quality changes in Chinese cabbage with storage period and temperature: A review. J. Biosyst. Eng. 41, 373–388. doi: 10.5307/JBE.2016.41.4.373

[B48] Tandayu E. Borpatragohain P. Mauleon R. Kretzschmar T. (2022). Genome-wide association reveals trait loci for seed glucosinolate accumulation in Indian mustard (Brassica juncea l.). Plants (Basel) 11 (3), 364–375. doi: 10.3390/plants11030364 35161346 PMC8838242

[B49] Vallone S. Sivertsen H. Anthon G. E. Barrett D. M. Mitcham E. J. Ebeler S. E.. (2013). An integrated approach for flavour quality evaluation in muskmelon (Cucumis melo l. reticulatus group) during ripening. Food Chem. 139, 171–183. doi: 10.1016/j.foodchem.2012.12.042 23561094

[B50] Wang Z. Y. (2020). QTL Mapping of Fructose Content and Functional Analysis of Hexose Transporter MdHT2.2 in Apple (Shanxi: Northwest Agriculture & Forestry University).

[B51] Wang Y. Feng C. Zhai Z. Peng X. Wang Y. Sun Y.. (2020). The apple microR171i-SCARECROW-LIKE PROTEINS26.1 module enhances drought stress tolerance by integrating ascorbic acid metabolism. Plant Physiol. 184, 194–211. doi: 10.1104/pp.20.00476 32680976 PMC7479918

[B52] Wang H. Wu J. Sun S. L. Liu B. Cheng F.. (2011). 'Glucosinolate biosynthetic genes in brassica rapa. Gene 487, 135–142. doi: 10.1016/j.gene.2011.07.021 21835231

[B53] Wang K. Zhang Z. Y. Tsai H. I. Liu Y. F. Gao J. Wang M.. (2021). Branched-chain amino acid aminotransferase 2 regulates ferroptotic cell death in cancer cells. Cell Death Differ. 28, 1222–1236. doi: 10.1038/s41418-020-00644-4 33097833 PMC8027606

[B54] Wiese A. Elzinga N. Wobbes B. Smeekens S. (2005). Sucrose-induced translational repression of plant bZIP-type transcription factors. Biochem. Soc. Trans. 33, 272–275. doi: 10.1042/BST0330272 15667324

[B55] Zang Y. X. Kim J. H. Park Y. D. Kim D. H. Hong S. B. (2008). Metabolic engineering of aliphatic glucosinolates in Chinese cabbage plants expressing arabidopsis MAM1, CYP79F1, and CYP83A1. Bmb Rep. 41, 472–478. doi: 10.5483/BMBRep.2008.41.6.472 18593532

[B56] Zang Q. L. Zhang Y. Han S. Y. Li W. F. Qi L. W. (2021). Transcriptional and post-transcriptional regulation of the miR171-LaSCL6 module during somatic embryogenesis in larix kaempferi. Trees 35, 145–154. doi: 10.1007/s00468-020-02026-2

[B57] Zuluaga D. L. Graham N. S. Klinder A. van Ommen Kloeke A. E. E. Marcotrigiano A. R. Wagstaff C.. (2019). Overexpression of the MYB29 transcription factor affects aliphatic glucosinolate synthesis in brassica oleracea. Plant Mol. Biol. 101, 65–79. doi: 10.1007/s11103-019-00890-2 31190320 PMC6695347

