# Supplementary material for: Identification of key genes controlling soluble sugar and glucosinolate biosynthesis in Chinese cabbage by integrating metabolome and genome-wide transcriptome analysis
Source: Front Plant Sci. 2022 Nov 25;13:1043489. doi: 10.3389/fpls.2022.1043489 (PMC9732556; doi:10.3389/fpls.2022.1043489)
Supplement: Supplementary file 1 [file DataSheet_1.docx]

Supplementary Material 1

# Supplementary Data

The RNA-seq data have been deposited in the NCBI Sequence Read Archive under accession number PRJNA867427.

# Supplementary Figures and Tables

## Supplementary Figure


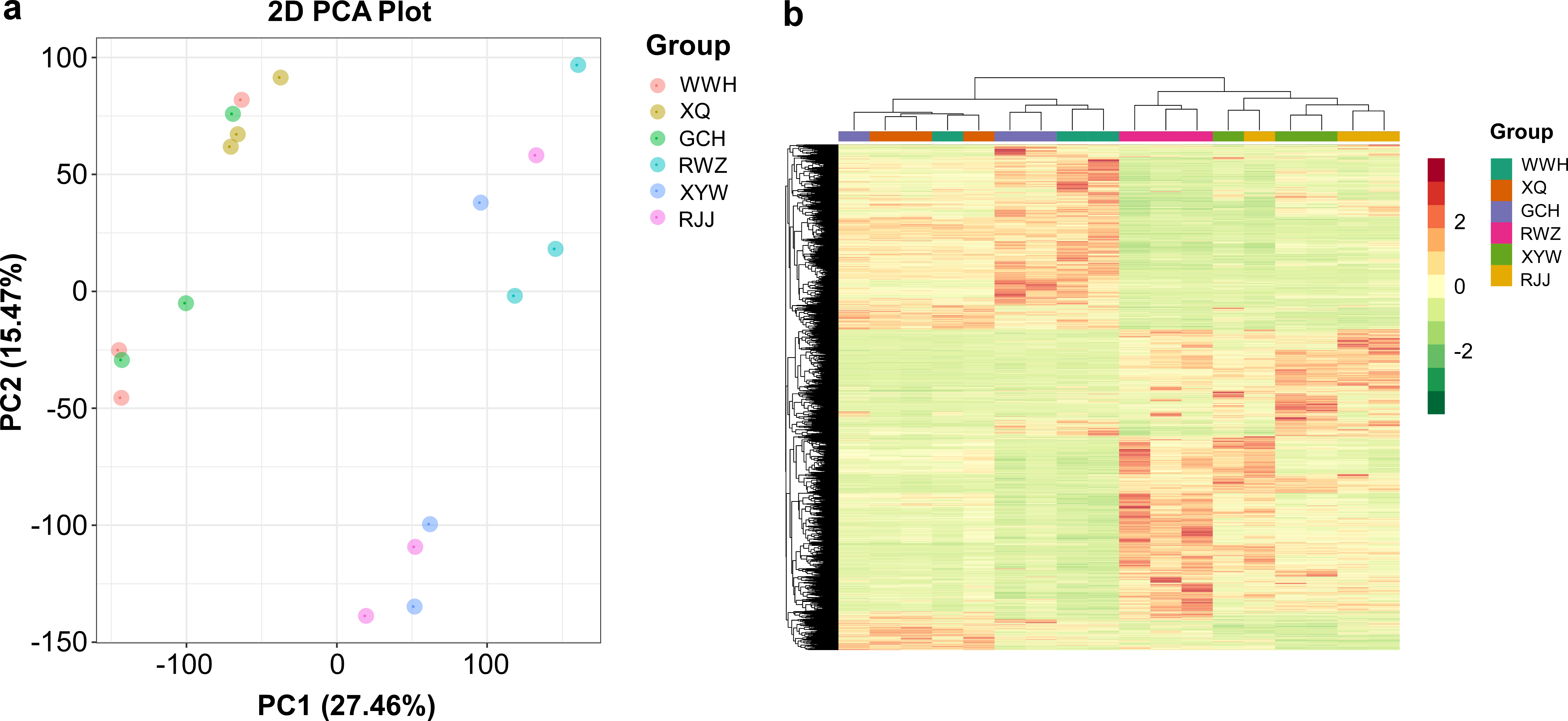


**Supplementary Figure 1.** The PCA and heatmap analysis of metabolites in Chinese cabbage with yellow and white inner-leaf color. (a) PCA score plot. (b) Clustering heatmap of all metabolites. Each sample was represented by a column, and each metabolite was represented by a row. The abundance of each metabolite was represented by a bar with a specific color.

**Supplementary Table 1. qRT-PCR Primer**

|  | ID |  | Sequence |  | ID |  | Sequence |
| --- | --- | --- | --- | --- | --- | --- | --- |
| 1 | BraActin-F | F | GGAGCTGAGAGATTCCGTTG | 18 | BraA03g017890 | F | TGCGGATATGAGCAGAGCCA |
|  | BraActin-R | R | GAACCACCACTGAGGACGAT |  |  | R | GTCTCCTGCAACTCCGCTTG |
| 2 | BraA04g001930 | F | GGTCTGCGTGCCGTACATAG | 19 | BraA04g030430 | F | GAGCACGAGAGAAGGTTGTAAG |
|  |  | R | GAAGGCTCGCGAAAGAGAGG |  |  | R | TACGACAAGCGGATGGTTATG |
| 3 | BraA02g036250 | F | CTGCAGTGGTGTCAGCTGTT | 20 | BraA06g034960 | F | GCCGTCGTGAATGCTTATCT |
|  |  | R | TGAACGTCAACTCCGCACTG |  |  | R | CTTCTCCCACCACAACTCTAAC |
| 4 | BraA05g028290 | F | GAATATGCAGGCCACGCTGA | 21 | BraA09g000240 | F | GTGCCACCGAGAAGAGTTAAT |
|  |  | R | TGGTGGCCTACACCTAGAGC |  |  | R | CGCAAGCCGTCTCTTTATCT |
| 5 | BraA06g006960.3 | F | GTTTAGCACCGGCACCTGAA | 22 | BraA05g000580 | F | CCAAACTCCTCGTCCATTTCT |
|  |  | R | AGGAGCTGCACGGACATACT |  |  | R | CTCTGCCTCTTCTGGCTATTT |
| 6 | BraA03g029330 | F | CGAGCTTGCGAACTCCATCA | 23 | BraA09g056920 | F | AATGACGCCGTCCAGATTAC |
|  |  | R | GAAGCGTTCCCTCCAAACCC |  |  | R | GAACCTCTTGTGCTCACTCTC |
| 7 | BraA01g033330 | F | TGCACAGCTGAAAGGCAACA | 24 | BraA01g038890 | F | GAGAGAGGAGCCAAGACCGT |
|  |  | R | TGTCAGTGAAGCCACGTTCG |  |  | R | TTGCGCTTGGCCATAGTCAG |
| 8 | BraA08g002520 | F | GCTGGGAGTCCAGTCAAACG | 25 | BraA02g011810 | F | TCCCTCTTAACCCGCTCCAG |
|  |  | R | CGATTCCACAGTGCCGACAT |  |  | R | CCCGGTGGGATGAAGCTAGA |
| 9 | BraA08g027290 | F | CACCCAAGCCAGCGGTTAAT | 26 | BraA02g042740 | F | ACCGGTTTCGAACCAGCAAA |
|  |  | R | TAAACGACAACGGCTGCGAA |  |  | R | TTGCGACCCGGTTAAGAAGC |
| 10 | BraA09g047460 | F | GCAGCCTACCTTAGGTGAAAT | 27 | BraA03g006050 | F | GTTTGGAGAATGGGCGGTGT |
|  |  | R | CTCCATACCAGCACTACCATTC |  |  | R | ATGCTCCCTGACTCATCAGC |
| 11 | BraA10g027720 | F | AAGGAGAAGTTGCTGGAAGAG | 28 | BraA06g015460 | F | GGCGGCAGCATGATAGACAA |
|  |  | R | GTCCTGGTGAGACATCCATATT |  |  | R | GTGGCTACCACTCCTTTGGC |
| 12 | BraA02g044910 | F | CTCTGGAGGACCAGCTCGAA | 29 | BraA06g018360 | F | AGCTCCCTCTCGCTCGTATC |
|  |  | R | CGGGAGAGTTTCCGGAGGAT |  |  | R | GTGGAGCCAAGAACGGATGG |
| 13 | BraA05g004800 | F | TCGGCGTCAATGTCGTTCTC | 30 | BraA07g000260 | F | GGTTTACGACACCGCCTGTT |
|  |  | R | CCGTCACGCTCAAAGGACAA |  |  | R | TGGCTGCATCTACGCTCTCT |
| 14 | BraA07g015190 | F | GGAGAGGCAGTGTCTCGCTA | 31 | BraA07g009390 | F | GCCAAGCCAAACTCCAGGAA |
|  |  | R | CCCTCCATTTCCCAGCAACC |  |  | R | GCCAAGCCAAACTCCAGGAA |
| 15 | BraA05g032790 | F | GTCACGAGCGAGCATCTCTG | 32 | BraA07g025950 | F | ACGCACCTTAGCCAAACGAG |
|  |  | R | AGCCTCGAACTCATCGTCCA |  |  | R | ACGCACCTTAGCCAAACGAG |
| 16 | BraA01g042050 | F | AGTATGGCCGCAACGACATC | 33 | BraA08g034430 | F | GTTCTTGATCCTGTCCCTCTTG |
|  |  | R | AGCTCCTTGTACCGCTCCTT |  |  | R | GCTTGCCTTTCTCTTTGATGAC |
| 17 | BraA09g014250 | F | TGTGGACGCTCATGTTTGGG |  |  |  |  |
|  |  | R | AGCAACACCCTGTCCCTTCT |  |  |  |  |

Note: “F” means forward, “R” means reverse.

**Supplementary Table 2. The detail of key transcription factors.**

| ID | Family | Category | Classification | Name |
| --- | --- | --- | --- | --- |
| BraA08g002520.3C | C2C2-GATA | TF | C2C2->C2C2-GATA | BraA08gGATA28 |
| BraA08g027290.3C | C3H | TF | C3H | BraA08gSOP1 |
| BraA09g047460.3C | bZIP | TF | bZIP | BraA09gAREB3 |
| BraA10g027720.3C | MADS-MIKC | TF | MADS->MADS-MIKC | BraA10gAGL25 |
| BraA02g044910.3C | MADS-MIKC | TF | MADS->MADS-MIKC | BraA02gAGL70 |
| BraA05g004800.3C | SNF2 | TR | SNF2 | BraA05gCHR10 |
| BraA07g015190.3C | GRAS | TF | GRAS | BraA07gSCL1 |
| BraA05g032790.3C | AP2/ERF-ERF | TF | AP2/ERF->AP2/ERF-ERF | BraA05gRAP2.2 |
| BraA01g042050.3C | SNF2 | TR | SNF2 | BraA01gCHR11 |
| BraA09g014250.3C | RWP-RK | TF | RWP-RK | BraA09gNLP6 |
| BraA03g017890.3C | SBP | TF | SBP | BraA03gSPL3 |
| BraA04g030430.3C | FAR1 | TF | FAR1 | BraA04gFRS5 |
| BraA06g034960.3C | MADS-M-type | TF | MADS->MADS-M-type | BraA06gAGL97 |
| BraA09g000240.3C | bZIP | TF | bZIP | BraA09gTGA1 |
| BraA05g000580.3C | WRKY | TF | WRKY | BraA05gWRKY23 |
| BraA09g056920.3C | MYB-related | TF | MYB->MYB-related | BraA09gMYB1R1 |
| BraA01g038890.3C | NF-YC | TF | NF-Y->NF-YC | BraA01gNF-YC11 |
| BraA02g011810.3C | GARP-ARR-B | TF | GARP->GARP-ARR-B | BraA02gARR18 |
| BraA02g042740.3C | MYB | TF | MYB->MYB | BraA02gMYB34 |
| BraA03g006050.3C | NAC | TF | NAC | BraA03gNAC41 |
| BraA06g015460.3C | C3H | TF | C3H | BraA06gC3H6 |
| BraA06g018360.3C | NF-YC | TF | NF-Y->NF-YC | BraA06gNF-YC4 |
| BraA07g000260.3C | GARP-G2-like | TF | GARP->GARP-G2-like | BraA07gGPRI1 |
| BraA07g009390.3C | bHLH | TF | bHLH | BraA07gbHLH77 |
| BraA07g025950.3C | C2H2 | TF | C2H2 | BraA07gZFP1 |
| BraA08g034430.3C | SNF2 | TR | SNF2 | BraA08gSNF2 |
